# The Role of Matrix Metalloproteinase in Inflammation with a Focus on Infectious Diseases

**DOI:** 10.3390/ijms231810546

**Published:** 2022-09-11

**Authors:** Han Sol Lee, Woo Joo Kim

**Affiliations:** 1 Asia Pacific Influenza Institute, Korea University College of Medicine, Seoul 02841, Korea; 2 Division of Infectious Diseases, Department of Internal Medicine, Korea University College of Medicine, Seoul 02841, Korea

**Keywords:** influenza A virus, SARS-CoV-2, matrix metalloproteinases, infectious diseases

## Abstract

Matrix metalloproteinases (MMPs) are involved in extracellular matrix remodeling through the degradation of extracellular matrix components and are also involved in the inflammatory response by regulating the pro-inflammatory cytokines TNF-α and IL-1β. Dysregulation in the inflammatory response and changes in the extracellular matrix by MMPs are related to the development of various diseases including lung and cardiovascular diseases. Therefore, numerous studies have been conducted to understand the role of MMPs in disease pathogenesis. MMPs are involved in the pathogenesis of infectious diseases through a dysregulation of the activity and expression of MMPs. In this review, we discuss the role of MMPs in infectious diseases and inflammatory responses. Furthermore, we present the potential of MMPs as therapeutic targets in infectious diseases.

## 1. Introduction

Matrix metalloproteinases (MMPs) are zinc-dependent endopeptidases that belong to the metzicin superfamily and are involved in the degradation and remodeling of extracellular matrix (ECM) components, such as fibronectin, laminin, collagens, elastin, and basement membrane glycoproteins. MMPs play important roles in physiological processes, such as wound healing, angiogenesis, embryonic development, synaptic plasticity, cell polarity, cell migration, and proliferation. In particular, the role of MMPs in angiogenesis, tumor invasion and metastasis mechanisms has been extensively studied. The expression of MMPs by tumors is closely associated with cancer metastasis [1,2]. Based on molecular biological studies, cancer researchers have postulated that MMP inhibitors would be effective against invasive cancer, and have, thus, conducted several clinical studies using various drug candidates [3,4]. Unfortunately, the severe side effects have resulted in the failure of several clinical trials. However, the failure of clinical trials using MMP inhibitors due to severe side effects underscores the diverse and sometimes paradoxically contradictory roles of MMPs in pathology, and the incompleteness of our knowledge of their biological functions in vivo. This is likely due to our historical over-reliance on in vitro models in MMP research. MMPs cleave ECM components, allowing signaling molecules and receptors to regulate cytokine signaling. Moreover, they disrupt the tissue–blood vessel barrier (TBB), and influx of infectious agents and immune cells during inflammation and infectious diseases. In this review, we provide an overview of the role of MMPs in infection-associated pathologies and discuss the therapeutic potential of MMP inhibitors in infectious diseases.

## 2. Matrix Metalloproteinase and Inflammatory Diseases

More than 24 MMPs identified in vertebrates have been classified as collagenases, gelatinases, stromelysins, elastases, and membrane-type MMPs (MT-MMPs), according to their substrates and structural differences. Most MMPs have multiple domains: a pre-domain (signal peptide), a pro-domain, a catalytic domain, and a C-terminal-hemopexin-like (HPX) domain. However, a few MMPs have other structural features. For example, gelatinases (MMP-2 and MMP-9) contain a fibronectin type II (FN) motif before the zinc-binding motif in the catalytic domain that allows binding to gelatin and collagen. Matrilysin (MMP-7 and MMP-26) and MMP-23 do not have a HPX domain. MMP-23 differs from the other MMPs in that it has specific domains such as a cysteine array, an Ig-like domain, and a signal anchor with a transmembrane domain that replaces the pre-domain. MT-MMPs have a glycosylphosphatidylinositol (GPI) anchor (MMP-17/MT4-MMP and MMP-25/MT6-MMP) or a transmembrane domain (MMP-14/MT1-MMP, MMP-15/MT2-MMP, MMP-16/MT3-MMP, and MMP-24/MT5-MMP). The pro-domain contains a cysteine thiol group that interacts with zinc bound by three histidines in the zinc-binding motif, and this interaction keeps the inactive state of MMPs (ProMMPs). Proteolytic cleavage of the pro-domain or modification of the cysteine thiol group activates the MMPs. Furthermore, reactive oxygen species (ROS) can activate MMPs through the oxidation of cysteine thiol groups of the pro-domain during inflammation [5,6]. Hypochlorous acid activates proMMP-7 by oxidizing cysteine thiol residues [5]. Peroxynitrite activates proMMP-1, -2, -8, and -9 by cysteinyl S-glutathiolation [7,8]. Generally, activated MMPs are regulated by four natural tissue inhibitors of metalloproteinases (TIMPs) that bind to the catalytic domain in a 1:1 non-covalent manner. An imbalance between MMPs and TIMPs often leads to inflammation and activates an immune response. The catalytic domain comprises a zinc-binding motif with three histidine residues and an active site with a glutamic acid residue. The catalytic domain determines the substrate specificity of the MMPs. The HPX domain is involved in substrate binding and modulates the activity of MMPs. TIMP-1 and TIMP-2 bind to the HPX domain of MMP-9 and MMP-2, respectively. Bannikov et al. [9] observed by in situ zymography that gelatinolytic activity correlates with the localization of proMMP-9 in human placenta, and furthermore demonstrated that proMMP-9 gains proteolytic activity when bound to a substrate in vitro even while retaining an intact propeptide, suggesting that proteolytic activation of MMPs may not be strictly necessary. These findings indicate that recombinant proMMP-9 containing the HPX domain can be used as an antagonist to modulate MMP-9 activity. The HPX domain of MMP-2 binds to monocyte chemotactic protein 3 (MCP-3, also known as CC-chemokine ligand 7, CCL7) in the yeast two-hybrid system. Moreover, MCP-3 is cleaved by MMP-2 [10]. Cleaved MCP-3 acts as a chemokine antagonist, thereby modulating the inflammatory response [10]. In addition, CXC-chemokine ligand 12 (CXCL12, also known as SDF-1α) is cleaved by MMPs and loses its binding affinity to CXCR4 [11]. Similarly, MMP-1, -3, and -13 cleave CCL2 (also known as MCP-1), CCL8 (MCP-2), and CCL-13 (MCP-4) and allow them to act similarly to chemokine antagonists [12]. In contrast, MMP-9 cleaves CXCL5 and CXCL8 to increase their chemoattractive properties, resulting in an efficient recruitment of neutrophils [13]. MMP-7 is involved in syndecan-1 cleavage, which releases CXCL1 to recruit neutrophils to the site of injury [14]. MMPs regulate the activation [15,16] and inactivation [15] of the pro-inflammatory cytokine, interleukin-1 beta (IL-1β). MMP-7 and MMP-12 also regulate tumor necrosis factor-alpha (TNF-α) levels. They cleave proTNF-alpha to release active TNF from macrophages [17,18]. 

In addition, MMPs are known to be secreted from cells, but also accumulate into cells through re-entry after secretion. Intracellular MMPs are activated through oxidative stress, post-translational modification, and proteolytic cleavage. Activated MMPs interact with intracellular molecules to regulate intracellular signaling processes [19]. For example, MMP-2, -9, and -13 bind to the LDL-associated protein-1 receptor and are re-entered by endocytosis. MMP-2 and -9 regulate gene expression by interaction with PARP-1 and histone H3 by translocating to the cell nucleus via nuclear localization sequences [19]. In particular, MMP-2, which is abundant in cardiomyocytes, is activated in the cytoplasm due to oxidative stress and enhances glycogen synthase kinase-3b activity, which may contribute to cardiac damage [19]. Furthermore, MMP-2 can induce cardiac contractile dysfunction by targeting several proteins, such as troponin I, α-actinin, myosin light chain-1, and junctophilin-2 [19]. In skeletal muscle fibers, intracellular MMP-2 is known to exist due to inefficient recognition of the N-terminal secretion signal, and little is known about its physiological function. Fallata et al. [20] demonstrated the presence of zebrafish MMP-2 within the myocytes of embryonic and adult skeletal muscle of zebrafish and suggested that it may play an important role in the physiology of striated muscle. Consequently, intracellular MMPs may be involved in pathological mechanisms by mediating cellular metabolism and signaling pathways. Moreover, secreted MMPs are involved in acute and chronic inflammation through cytokine and chemokine modulations and can act as a switch in inflammation-related diseases and their regenerative phase. 

### 2.1. Matrix Metalloproteinase and Lung Diseases

MMPs play an important role in the progression of chronic inflammatory diseases, including pulmonary fibrosis (PF), chronic obstructive pulmonary disease (COPD), and coronary heart disease such as atherosclerosis [19,21]. The development of emphysema-associated COPD and PF are associated with the deposition of ECM in the lungs. The expression of MMP-1, MMP-2, MMP-8, MMP-9, MMP-12, and MT1-MMP in the lungs are upregulated in patients with COPD [22,23,24,25,26,27]. MMP-12 and other MMPs cleave elastin and other ECM components, causing the lung to lose its elastic recoil. Moreover, cleaved elastin fragment and collagen-derived peptide Pro-Gly-Pro mediate monocyte infiltration and promote inflammation, thereby prolonging the inflammatory response and ECM disruption. Interestingly, MMP-12 deficient mice exposed to cigarette smoke do not develop emphysema and exhibit reduced macrophage recruitment to the lung [28,29,30,31]. MMP-12 is rarely detected in healthy macrophages, whereas its expression is increased in alveolar macrophages of smokers with COPD [32]. These findings indicate that MMP-12 plays a pivotal role in emphysema-associated COPD. The increase in MMP-1, MMP-2, MMP-8, and MMP-9 expression levels were found in sputum and bronchoalveolar lavage (BAL) from patients with asthma or COPD [33]. McKeown et al. measured and analyzed the expression of MMPs in the BAL of patients with idiopathic pulmonary fibrosis (IPF) (n = 20) and healthy individuals without respiratory disease (n = 8) using Liminex array [34]. In the BAL of patients with IPF, MMP-2, MMP-3, MMP-7, MMP-8, and MMP-9 expression levels were markedly upregulated compared with those in the healthy control. MMP-3 deficient mice are protected against bleomycin-induced PF, and the overexpression of recombinant MMP-3 in rat lung leads to myofibroblast accumulation and PF [35]. In addition, Wnt/beta-catenin signaling, which is important in the pathogenesis of IPF, is activated by MMP-3 [36,37,38]. In damaged lung epithelial cells, MMP-7 induces the release of the syndecan-1/CXCL-1 complex to promote fibrosis through neutrophil influx. Subsequently, MMP-7 acts as an anti-fibrotic mediator to induce immunosuppressive leukocyte influx to resolve the fibrotic condition [39]. Decreased PF and increased protein expression of macrophage inflammatory protein-1 alpha (MIP-1α) and CXCL10 (also known as IP-10) were observed in MMP-8 deficient mice [40]. CXCL10 exhibits anti-fibrotic activity through inhibition of fibroblast chemotaxis. MMP-9 expression level is elevated in both humans and experimental lung fibrosis [41,42], but animal studies using knockout mice showed conflicting results for the role of MMP-9, making it difficult to demonstrate its role clearly [43,44]. Although the role of a few MMPs is difficult to establish clearly, clinical and in vitro studies consistently support the association of MMPs with chronic lung disease.

### 2.2. Matrix Metalloproteinase and Cardiovascular Diseases

In cardiovascular diseases, MMPs are activated through a variety of pathways to influence the process of atherosclerotic lesion formation. Thrombin exerts a potent pro-inflammatory effect on vascular cells, upregulates MMP-10 expression, and promotes MMP-2 activation [45,46]. The cathepsins present in atherosclerotic lesions proteolytically activate MMPs, including MMP-1, MMP-2, and MMP-9 [47,48,49,50,51]. Hormonal stimulation using angiotensin II (Ang II) and estrogen also activates vascular MMPs, including MMP-2, MMP-8, MMP-9, MMP-13, and MT1-MMP [52,53,54,55,56,57]. In addition, oxidative stress and inflammatory cytokines, such as TNF-α, IL-6, IL-1β and IL-18, activate MMPs and are involved in vascular remodeling [58,59,60]. In vitro studies have shown that eNOS gene transfected smooth muscle cells (SMCs) reduce MMP-2 and MMP-9 expression and impair SMC migration. Furthermore, nitric oxide induces direct activation by chemical modification of thiols in the cysteine switch of proMMP-2 [7,8,58]. TNF-α signaling activates transcription of the MMP-9 gene by upregulating nuclear FoxO4. MMP-9 plays a major role in vascular SMC migration during restenosis and atherosclerotic lesions [59]. IL-18 mediates NF-κB and AP-1 activation and MMP-9 expression to induce human coronary artery SMC migration [60]. The plasma levels of MMP-1, MMP-7, MMP-8, MMP-9, and MMP-10 are elevated in patients with coronary artery disease (CAD), whereas those of MMP-2 and MMP-3 levels are decreased [61,62,63]. Moreover, the expression and activity of MMP-1, MMP-8, and MMP-13 are increased in human atherosclerotic plaques, resulting in plaque instability through collagen degradation [64,65,66,67,68]. Studies of inhibitor-treated or MMP-13-deficient mice show that MMP-13 has no effect on plaque size, but is involved in collagen degradation in plaques [65,69]. MMP-12 is expressed by macrophages present in advanced plaques and contributes to necrotic core expansion by promoting macrophage apoptosis [70,71]. MT1-MMP is also most prominently expressed in foam cell macrophages of advanced plaques, thereby promoting plaque progression and instability [72,73]. MMP-1, MMP-8, MMP-12, MMP-13, and MT1-MMP not only degrade the extracellular matrix proteins present in the fibrous cap, but also recruit monocytes/macrophages to the plaque region to promote foam cell formation and death. As a result, plaque instability is increased by MMP-mediated enhancement of lipid core expansion, formation of plaque thrombus, and thinning of the fibrous cap. In contrast, MMP-2, MMP-3, and MMP-9 are involved in promoting plaque stability [74]. Increased fibrous layer and plaque size in MMP-3/ApoE double knockout mice suggest that MMP-3 is involved in plaque stability [75]. Increased expression levels of MMP-2 and MMP-9 were confirmed in the expansively remodeled plaques of patients who died from coronary artery disease [61,76,77,78]. These results suggest that MMP-2, MMP-3, and MMP-9 can promote the migration of vascular smooth muscle cells, stabilizing atherosclerotic plaques. Furthermore, the absence of TIMP-1 in mice increases neointimal formation [79]. Recent studies have reported that regulating MMPs and TIMP expression via specific microRNAs has a direct effect on plaque progression [80,81]. In atherosclerosis, MMPs act as regulators of plaque stability and progression, and the expression of these MMPs is regulated by cytokines and oxidative stress. Acute inflammation is triggered by a variety of factors, including pathogens infection, smoking, tissue injury and toxic compounds. The healthy inflammatory response functions to remove causes of inflammation triggered by environmental insults and pathogens, restoring tissue homeostasis. 

## 3. Matrix Metalloproteinase and Infectious Diseases

Infectious diseases are characterized by the various symptoms resulting from infection by microorganisms, such as bacteria, viruses, protozoa, and fungi. Excessive activity of MMPs induced by infection has been implicated in the pathogenesis of infectious diseases, such as sepsis, meningitis, tuberculosis, Lyme disease, and pneumonia.

### 3.1. Bacterial Pneumonia

Pneumonia is a lung infection caused by pathogens, including *Pseudomonas aeruginosa*, *Streptococcus pneumoniae*, *Haemophilus influenzae*, *Staphylococcus aureus*, influenza viruses, adenoviruses, and respiratory syncytial viruses. Epithelial cells not only act as a physical barrier, but also regulate inflammation by secreting MMPs and cytokines to recruit inflammatory cells to attack the invasive pathogens. Bacterial infection induces the expression of MMP-7 and MMP-10 in bronchial epithelial cells [82,83,84]. qRT-PCR and microarray analyses confirmed that mRNA expression levels of MMP-7 and MMP-10 were increased in the airway epithelium of mice infected with *P. aeruginosa* [82]. Further, *P. aeruginosa* flagellin increases the mRNA and protein expression levels of MMP-7 in human lung epithelial cells [83]. Since MMP-7 promotes the development of PF and induces leukocyte influx, the increased expression of MMP-7 by *P. aeruginosa* is thought to be related to its pathology. MMP-28 mRNA level is down-regulated in the lungs of *P. aeruginosa* infected mice [84]. In contrast, MMP-28 mRNA expression is increased in bone marrow-derived macrophages stimulated by bacteria, but not in alveolar macrophages [84]. Compared with that in wild-type (WT) mice, *P. aeruginosa* infection accelerated macrophage influx, improved bacterial clearance, and reduced pulmonary neutrophilia in MMP-28 knockout mice [84]. Additionally, M2 polarization and bleomycin-induced lung fibrosis were reduced in MMP-28 knockout mice [85]. These findings indicate that MMP-28 can modulate the inflammatory response through macrophage activation and neutrophil recruitment. 

MMP-8 and -9 are increased in patients with hospital-acquired pneumonia (HAP), and their concentrations in mini-BAL fluid are positively correlated with clinical severity [86]. MMP-8 and MMP-9 levels are increased in the lungs of patients with HAP who are infected with high-risk pathogens. Moreover, MMP-9 activity is increased in such patients compared with that in the control and low-risk pathogen-infected patients [87]. MMP-8 and MMP-9 are produced by neutrophils or macrophages [88], and are involved in neutrophil migration [89]. MMP-9 and MMP-2 play an important role in the migration of various leukocytes. MMP-9 increases the influx of immune cells through destruction of basement membrane components [88,90]. However, Rosendahl et al. suggested that bacteria utilized MMP-9 to self-propagate at local sites of infection by increasing TBB permeability [91]. The increase in TBB permeability by MMPs, including MMP-9, is thought to affect two aspects of inflammatory cell influx and pathogen dissemination in the pathology of pneumonia. MMP-9 increase is observed not only in patients with HAP, but also in those with community-acquired pneumonia (CAP) and ventilator-associated pneumonia [92,93]. MMP-9/TIMP-1 ratio and protein expression levels are significantly increased in patients with CAP compared with healthy control. Furthermore, plasma TIMP-1 level is positively correlated with CAP severity [94]. The balance of MMP-9/TIMP-1 and the activity of MMP-9 appear to play important roles in the pathology of bacterial pneumonia. The role of MMP in pneumonia caused by viral infection, including influenza A virus (IAV), will be described later.

### 3.2. Bacterial Sepsis

Sepsis is a disease in which pathogens or endotoxins cause a systemic inflammatory response throughout blood vessels, resulting in major organ dysfunction. Various clinical studies have shown increased concentrations of MMPs and TIMPs, including MMP-1, MMP-2, MMP-3, MMP-7, MMP-8, MMP-9, TIMP-1, TIMP-2, and TIMP-3, in the plasma of patients with sepsis [95,96,97,98]. However, statistical studies on the association between an increase in MMP and disease severity have shown that MMP-7 and MMP-9 were negatively correlated with disease severity and MMP-3 and TIMP-1 concentrations were associated with mortality [95,96,97]. Additionally, serum levels of MMP-8 and TIMP-1 were correlated with mortality in patients with sepsis [98,99]. Various animal and in vitro studies were performed to demonstrate the role of MMPs in clinically associated symptoms with sepsis severity. In the animal model of sepsis, MMP-2 and MMP-9 were activated, blood-brain barrier (BBB) permeability increased, and BBB permeability was reversed by MMP inhibitor [100]. Additionally, abdominal sepsis in MMP-9 deficient mice showed that MMP-9 deficiency reduces leukocyte recruitment [101]. Tressel et al. reported that proMMP-1 and active MMP-1 were increased in plasma of patients with sepsis, and active MMP-1 level directly correlated with mortality [102]. In addition, MMP-1 is considered to be an important activator of PAR1, and the blockage of MMP-1 activity inhibits endothelial barrier disruption, lung vascular permeability, and cytokine storm [102,103]. In a study of a cecal ligation and puncture-induced sepsis mouse model, Solan et al. reported that MMP-8 deficiency improved the survival of mice and decreased early lung neutrophil infiltration, and cytokine and chemokine expression [99]. The inflammatory response by MMP-8 was suggested to be directly related to NF-κB activity [99]. MMP-13 is an important mediator regulating the intestinal epithelial barrier integrity by modulating TNF-α activity in sepsis and inflammatory bowel disease [104]. These studies provide evidence that the MMPs increased during sepsis are involved in promoting inflammation and disease severity. Unlike these MMPs, MMP-7 expression in the small intestine is involved in antimicrobial and homeostasis maintenance through α-defensin activation. In mice, MMP-7 deficiency impairs defense against gut pathogens such as *Salmonella typhimurium* and *Escherichia coli* [105]. In clinical and animal studies, MMP-7 is considered to be more important in eliminating infectious agents through immune activation in sepsis patients. However, some controversies have been noted. The absence of MMP-7 protects mice from LPS-induced lethality due to a decrease in α-defensin activation and the subsequent inhibition of IL-6 secretion by macrophages [106]. This finding indicates that MMP-7 can exhibit pro-inflammatory activity through α-defensins activity. 

### 3.3. Other Bacterial Infectious Diseases

*Borrelia burgdorferi*, which causes Lyme disease, upregulates the release and activation of MMP-9 and MMP-1 in human monocytes. Furthermore, activated MMPs may be key factors in the transmission of the Lyme disease spirochete [107,108]. MMP-13 expression level is increased in bacteria-induced periodontitis and the inhibition of MMP-13 expression decreases bone resorption and the production of inflammatory mediators [109,110,111]. In neuroinflammatory diseases such as bacterial meningitis, MMPs open the BBB and facilitate the influx of blood-derived immune cells [112,113,114]. In particular, high levels of MMP-8 and MMP-9 are found in the cerebrospinal fluid of patients with bacterial meningitis [115].

The induction of MMP expression and subsequent activation of proteolytic activity resulting from inflammation and the immune response is central to the pathology of many bacterial infections. In particular, it is involved in the inflammatory response and clearance of pathogens through the regulation of the TBB permeability involved in the influx of immune cells and the movement of the pathogen, owing to the regulation of the expression of MMPs by the infection. In addition, these results are not limited to bacterial infections, but are also observed in viral infections. Recently, correlations of MMPs with viral pathologies, including influenza A virus, Zika virus, dengue virus and severe acute respiratory syndrome coronavirus 2 (SARS-CoV-2), have been reported [116,117,118,119,120,121,122,123,124,125,126,127,128,129,130,131,132,133,134,135,136,137,138,139,140,141,142,143,144,145,146,147,148,149,150,151].

## 4. Virus-Induced Matrix Metalloproteinases Expression and Pathogenesis

### 4.1. Zika Virus Infection

Zika virus (ZIKV) belongs to the *flavivirus* family, which includes the dengue virus (DENV), Japanese encephalitis virus (JEV), and West Nail virus. ZIKV is transmitted through insects including mosquitoes and ticks. ZIKV was discovered in 1947, but has recently spread significantly in South America. ZIKV causes microcephaly and Guillain-Barré syndrome; and various studies have been conducted to elucidate the underlying causes and mechanisms [116]. In a study examining the concentrations of cytokines, MMP-2 and MMP-9 levels remained high in the semen and plasma of ZIKV-infected patients, even after the virus was cleared [117]. Additionally, MMP-2 and MMP-9 levels were increased in the placenta of ZIKV-infected patients. This might have induced collagen degradation, thereby leading to placental villi immaturity [118]. In in vivo and in vitro studies, MMP-9 expression and activity were increased during ZIKV infection and the ZIKV non-structural protein 1 interacted with MMP-9 and promoted its K63-linked polyubiquitination to prevent proteosome degradation. Increased blood–testis barrier permeability, owing to increased MMP-9 stability and expression, led to the entry of ZIKV into the testis [119,120]. In addition, MMP-10 and MMP-13 activities were increased upon ZIKV infection of HUVEC cells, a human umbilical vein endothelial cell line [121]. These studies suggest that MMPs may be involved in the infection-induced placental damage and sexual contact infection. However, these results are insufficient to determine whether the ZIKV-induced MMP expression is involved in the induction of microcephaly. 

In particular, MMP-2 and MMP-9 levels are increased by ZIKV as well as by DENV and JEV infections to induce vascular leakage, in addition to increasing the BBB permeability [122,123,124]. A recent review on the role of MMPs in the pathogenesis of DENV highlighted a correlation between disease severity and MMP-2 and MMP-9 expression levels [125]. In the study of the association of MMPs with various diseases such as vascular disease, lung disease, and cancer, MMP-2 and MMP-9 have been extensively studied due to their broad substrate specificity.

### 4.2. Severe Acute Respiratory Syndrome Coronavirus 2 Infection

COVID-19, a disease caused by severe acute respiratory syndrome coronavirus 2 (SARS-CoV-2) infection through the respiratory tract, presents respiratory symptoms similar to those of influenza and pneumonia, and also affects other parts of the body [126]. In addition, sequelae may appear for a long time after recovery from COVID-19. One of the causes of COVID-19 sequelae is lung injury and multiple organ failure due to cytokine storm. MMPs play an important role in lung physiology. In clinical studies, plasma levels of MMP-2, MMP-3, and MMP-9 were associated with the severity of patients with COVID-19 [127,128]. Interestingly, MMP-2 level was decreased in the plasma of patients with COVID-19 compared with that of the control group [127]. Additionally, plasma level of MMP-2 was upregulated in patients with COVID-19 and hypertension, although still less than in healthy controls [127]. Elevation of MMP-2 in hypertension has been proved by several studies [54,128], but studies on the mechanism of MMP-2 decrease in patients with COVID-19, are still lacking. Upregulated MMP-9 induces leukocyte recruitment in lung diseases including COPD and asthma [34,88,89,90]. Circulating leukocyte count, IL-6, and myeloperoxidase levels were positively correlated with MMP-9 concentration in patients with COVID-19 [129], which suggests that MMP-9 is associated with inflammation in patients with COVID-19. MMP-3 plays a role in the pathogenesis of acute inflammation-induced lung injury, as evidenced by a reduction in the extent of lung injury via the inhibition of MMP-3 in animal models [130,131]. The increase in MMP-3 serum level in patients with COVID-19 was not observed after one week of hospitalization, which indicates that the MMP-3 activity contributes largely in the early stages of lung inflammation caused by COVID-19 [129]. Recently, the expression of MMPs in the lung biopsy samples of patients with COVID-19 and the mechanism of lung disease pathogenesis were reported [132]. MMP-2, MMP-7, MMP-8, and MT1-MMP were increased in the lungs of patients with COVID-19 [132]. In particular, MMP-2 and MMP-8 were significantly increased, indicating a positive correlation with soluble human leukocyte antigen G (HLA-G) and the triggering receptor expressed on myeloid cells 1 levels, which regulate an innate immune response [132]. Moreover, MMP-2 level in tracheal aspirate fluid is positively correlated with malondialdehyde level, which is an oxidative stress marker [132]. MMP-2/MMP-8 activity is involved in the lung pathogenesis of patients with COVID-19 by increasing the influx of immune cells into the lungs, secreting HLA-G, and regulating the immune response through oxidative stress [132]. Consequently, these findings suggested that various inflammatory response-related factors, including MMP activity, and oxidative stress, are involved in the lung pathogenesis of patients with COVID-19. In the SARS-CoV-2 challenge study using K18-hACE2-transgenic mice, MMP-8, -9, and MT1-MMP expression levels were increased in the lungs of mice, and lung damage and extensive leukocyte infiltration were observed [133]. In clinical and mouse studies, expression levels of MMP-8 and MT1-MMP were increased in SARS-CoV-2 infected lungs. The infiltration of immune cells into the lung tissue is thought to be increased by MMP-8, which is increased owing to SARS-CoV-2 infection. However, although MT1-MMP is known to be increased in patients with IPF [134], its role is unclear. A recent study on MT1-MMP knockout mice suggested that MT1-MMP increases in response to the potent pro-fibrotic microenvironment of, and is involved in the anti-fibrotic mechanism [135]. Unlike MMP-8, MT1-MMP is considered to be increased by a feedback loop in lung disease caused by SARS-CoV-2 infection. The regulation of MMPs in infectious diseases can appear as a response to inflammation and tissue damage, as well as direct regulation by infectious agents. Therefore, the pathological features of COVID-19, including severe lung disease and excessive inflammatory response, is hypothesized to be related to MMPs, and, therefore, MMPs inhibitors have the potential to be used to treat COVID-19 effectively. Although the effect of specific MMP inhibitors in alleviating the severity of COVID-19 has not yet been reported, there are reports that doxycycline, a non-specific MMP inhibitor, can reduce the need for intensive care unit hospitalization of patients with COVID-19 [136]. However, doxycycline is a broad-spectrum tetracycline antibiotic used for the treatment of bacterial and parasitic infections, and, therefore, cannot be regarded as an MMP-specific inhibitor.

### 4.3. Influenza A Virus Infection

Regarding the respiratory syncytial virus (RSV) and influenza A virus (IAV), that cause lung disease, MMP inhibition has been reported to reduce the severity of the viral pathologies [137,138,139,140]. In particular, IAV infection causes lung damage and increases the risk of various diseases, such as bacterial pneumonia, sepsis, and exacerbation of cardiovascular diseases. Various studies have demonstrated the mechanisms by which MMPs, particularly MMP-9, increase the risk of diseases caused by IAV infection [139,141,142,143,144]. For example, viral infection increases the expression of pro-inflammatory cytokines such as TNF-α, IL-6, and IL-1β, and increases the activity of the activator protein-1, NF-κb, and MAPK signaling pathways [117,121,124,133,138]. Subsequently, proteases including MMPs and trypsin are upregulated, resulting in the increased permeability and immune response. In particular, cytokine storms are often induced by uncontrolled neutrophil influx and alveolar macrophage activation. TNF-α, which is increased during IAV infection, induces MMP-9 secretion in neutrophils, causing the ECM collapse and resulting in an increase in the influx of immune cells to the site of infection, thereby increasing the severity of IAV infection. Furthermore, studies using MMP-9 deficient mice demonstrated the role of MMP-9 in lung diseases caused by IAV infection [144]. Lung injury was reduced in MMP-9 deficient mice owing to decreased immune cell influx and type I IFN levels compared with that in WT mice during H1N1 infection. Interestingly, studies on lung damage caused by secondary bacterial infections following influenza virus infection have reported that the MMP inhibitor, batimastat, reduced the inflammatory response and lung damage in the infected mice. In addition, Talmi-Frank et al. reported that the combination therapy of anti-MT1-MMP and the antiviral agent Tamiflu significantly increased ECM protection and survival rate from sepsis caused by *S. pneumoniae* [145]. These results demonstrate that MMP-9 and MT1-MMP play an important role in sepsis or pneumonia, and secondary bacterial infections caused by influenza virus infection.

IAV infection increases the risk of underlying diseases, including cardiovascular disease and hypertension, as well as lung disease. In particular, acute cardiovascular disease exacerbation due to influenza virus infection, and vaccination-mediated decrease in the risk of infection, have been reported. Cardiovascular disease is exacerbated during influenza virus infections due to increased immune cell infiltration and thrombosis through an unknown mechanism [146]. We predicted that MMPs may play an important role in the mechanism of IAV-induced cardiovascular disease exacerbation, and we demonstrated that IAV-induced MMP-13 expression in atherosclerosis plaques reduced plaque stability [147]. Cellular sources of MMPs in atherosclerosis plaques include endothelial cells, vascular smooth muscle cells, and macrophages. In particular, macrophages secrete various MMPs. We confirmed that MMP-13 was significantly increased in IAV-infected macrophages in vitro and in vivo. MMP-13 is mainly involved in plaque stability through collagen degradation. MMP-9 and MT1-MMP not only increase the influx of immune cells by increasing the permeability in IAV-infected lungs, but they may also induce the penetration of IAV-exposed macrophages to the atherosclerotic plaques. Virus-exposed macrophages increase MMP-13, contributing to decreased plaque stability and risk of rupture. Few studies have suggested a relationship between other MMPs and IAV, but this needs to be verified by further studies [148,149,150]. Additionally, a study on MMP inhibitor showed that treatment of doxycycline in IAV-infected mice reduced acute lung injury and decreased MMP-2/MMP-9, but did not change viral concentrations [151]. Moreover, pulmonary injury was reduced by the MMP inhibitory effect of doxycycline.

Altered MMP expression levels and activity in cells infected with viruses, including tick-borne encephalitis virus or varicella zoster virus (Table 1), are associated with the disease pathology [152,153,154,155,156,157,158,159,160,161,162,163,164,165,166,167,168,169,170,171,172,173,174,175]. Additional studies are needed to demonstrate the regulatory mechanism used during viral infections and its role in disease pathology. However, previous studies confirm that MMP expression is increased in various virus infections and that it is involved in the pathological mechanisms [116,117,118,119,120,121,122,123,124,125,126,127,128,129,130,131,132,133,134,135,136,137,138,139,140,141,142,143,144,145,146,147,148,149,150,151,152,153,154,155,156,157,158,159,160,161,162,163,164,165,166,167,168,169,170,171,172,173,174,175].

## 5. Conclusions and Future Directions

Microbial infection induces inflammation at the site of infection, leading to various diseases, such as pneumonia, meningitis, hepatitis, and cardiovascular disease. Various studies have demonstrated the involvement of MMPs in the development of these diseases and inflammation [19,176]. MMP-8 and MMP-9 levels are increased in patients with pneumonia, and this increase in lung tissue induces neutrophil influx, thereby contributing to the elimination of infectious agents. However, the increase in TBB permeability by MMP-9 may contribute to the spread of infectious agents as well as neutrophil influx. MMP-9 expression is significantly increased by infection with respiratory viruses, including IAV, SARS-CoV-2, HRV, and RSV, and is positively correlated to the severity of the diseases. Furthermore, IAV infection in MMP-9-deficient mice decreases the virus burden, lung injury, and increases adaptive immune response, which supports the pathological function of MMP-9 [144]. A recent report confirmed that MT1-MMP inhibition reduces the mortality and tissue damage caused by IAV infection, and also reduces secondary bacterial infections [145]. MT1-MMP activates proMMP-9 [177], suggesting that proMMP-9 activation may be reduced by MT1-MMP inhibition. In addition, increased BBB permeability by MMP-9 plays a pivotal role in infectious encephalitis and meningitis [123,124,142]. The symptoms caused by SARS-CoV-2 and IAV infection were alleviated by doxycycline and batimastat, which are broad-spectrum MMPs inhibitors [136,144,151]. MMPs, including MMP-2, MMP-3, and MMP-8, are involved in the pathogenesis of infectious diseases [86,87,88,95,96,97,98,115,127,148,149,150]. However, the therapeutic effect of inhibition of MMP-9 and MT1-MMP have only been demonstrated in animal models [145]. 

Many pathogens cause inflammatory responses in various cells, and these inflammatory responses are regulated by MMP expression and activation [7,8,9,10,11,12,13,14,15,16,17,18,19]. The regulation of MMPs expression and activation is regulated by various mediators. First, the transcription of MMPs by cells in infected tissues is regulated by pathogen and intracellular MMPs through the regulation of cell signaling pathways, including MAPK, NF-κb, and AMPK [19,178,179]. MMP-3 and MMP-12 are also translocated to the nucleus to modulate NK-κb signaling [19]. In particular, nuclear MMP-3 can regulate connective tissue growth factor (CTGF) expression by interaction with the heterochromatin protein gamma, and CTGF increases the transcription of several MMPs, including MMP-1, -2, -3, and -9, in cancer cells. These findings indicate that MMPs can be increased by a feedback loop. In addition, the activity of MMPs can be modulated by bacterial proteases and host serine protease [180,181]. For example, *P.aeruginosa* elastase, *Vibrio cholerae* protease, and thermolysin activate proMMP-1, -8, and -9 [180]. The regulatory mechanism of MMPs in infectious diseases is difficult to elucidate clearly, because it is related to various factors such as pathogen, reactive oxygen species, and intracellular molecules. Therefore, since many studies on MMPs are based on pathological studies, this review has focused on the pathological content.

In conclusion, MMPs are regulated by pathogen infections and are involved in the pathogenesis of infection, and the regulation of over-activated MMPs can alleviate a disease. Although there have been various clinical studies on MMP inhibitors, most MMP inhibitors have been clinically tested for cancer, but several clinical trials have failed due to the side effects. Therefore, it is suggested that MMP inhibitors may be more effective for inflammation caused by acutely applicable infectious diseases and injuries than for cancer with continuous use of therapeutic agents and side effects. Alleviation of pathological mechanisms through MMP inhibition in infectious diseases has been confirmed in some animal experiments, providing a proof-of-concept for MMP inhibitors. Although the therapeutic potential of MMP inhibitors in infectious diseases has been suggested, further studies including identification of specific targets and demonstration of effectiveness in clinical trials are needed. 

## Figures and Tables

**Table 1:** Viral infections in which matrix metalloproteinases activity is associated with pathology.

Virus	MMPs Regulation	References
Human Immunodeficiency Virus (HIV)	MMP-2 ↑, MMP-9 ↑, TIMP-1 ↓	[152,153,154,155,156]
Human T-Lymphotropic Virus type 1 (HTLV-1)	MMP-3 ↑, MMP-9 ↑	[157,158,159]
Hepatitis B Virus (HBV)	MMP-2 ↑, MMP-9 ↑, MT1-MMP ↑	[160,161,162]
Hepatitis C Virus (HCV)	MMP-2 ↑, MMP-9 ↑	[163,164]
Dengue Virus (DENV)	MMP-2 ↑, MMP-9 ↑	[125,165,166]
Japanese Encephalitis Virus (JEV)	MMP-2 ↑, MMP-7 ↑, MMP-9 ↑, TIMP-1 ↑↓, TIMP-2 ↑, TIMP-3 ↑	[123,167,168]
Respiratory Syncytial Virus (RSV)	MMP-2 ↑, MMP-9 ↑, MMP-10 ↑, MMP-12 ↑	[140,169,170,171]
Varicella-Zoster Virus (VZV)	MMP-2 ↑, MMP-3 ↑, MMP-8 ↑, MMP-9 ↑, MMP-12 ↑	[172,173]
Human Rhinovirus (HRV)	MMP-2 ↑, MMP-9 ↑	[174,175]

MMPs: matrix metalloproteinases.

## Data Availability

Not applicable.
